# Specificity and disease in the ubiquitin system

**DOI:** 10.1042/BST20150209

**Published:** 2016-02-09

**Authors:** Viduth K. Chaugule, Helen Walden

**Affiliations:** * MRC Protein Phosphorylation and Ubiquitylation Unit, College of Life Sciences, University of Dundee, Dow Street, DD1 5EH, U.K.

**Keywords:** ubiquitin, E3 ligase, RING, RBR, Fanconi anemia, Parkin

## Abstract

Post-translational modification (PTM) of proteins by ubiquitination is an essential cellular regulatory process. Such regulation drives the cell cycle and cell division, signalling and secretory pathways, DNA replication and repair processes and protein quality control and degradation pathways. A huge range of ubiquitin signals can be generated depending on the specificity and catalytic activity of the enzymes required for attachment of ubiquitin to a given target. As a consequence of its importance to eukaryotic life, dysfunction in the ubiquitin system leads to many disease states, including cancers and neurodegeneration. This review takes a retrospective look at our progress in understanding the molecular mechanisms that govern the specificity of ubiquitin conjugation.

## Introduction

Ubiquitination is a reversible post-translational modification (PTM) that affects the fate, function or localization of the modified protein. The conjugation of the 76-amino acid ubiquitin polypeptide requires sequential action of activating enzymes (E1s), conjugating enzymes (E2s) and ligase enzymes (E3s) resulting in an isopeptide link between the C-terminus of ubiquitin and a specific lysine on the target protein [1,2]. The attached molecule can support further building of chains from any of the seven lysines present on the surface of ubiquitin or its N-terminus, thus providing substantial signal diversity. Eukaryotic cells exploit different ubiquitin signals to modulate crucial homoeostatic processes. For example, at the onset of anaphase, Lys^48^-linked polyubiquitin signals on Securin triggers its proteolysis to induce chromosome segregation events [3]. In contrast, when damaged DNA stalls replication, a monoubiquitin signal on Lys^164^ of the proliferating cell nuclear antigen (PCNA) prompts recruitment of specialized polymerases that allow the replication machinery to bypass the damage [4,5]. In addition to the signal properties, the addition of just a single ubiquitin molecule alters the physicochemical features of the substrate surface [6].

Ubiquitin itself harbours different functional surfaces, for example the Ile^44^-hydrophobic patch (Leu^8^/Ile^44^/His^68^/Val^70^), which supports crucial non-covalent interactions during ubiquitination and signal recognition [7]. In polyubiquitin chains, the repetition of these surfaces results in localized signal amplification. Seven distinct ubiquitin chain types have been structurally characterized revealing remarkable topological differences and dynamic polymer conformations [8–17]. The distinct surfaces of ubiquitin are ‘read’ by an array of ubiquitin-binding domains (UBDs, ∼20 types) encoded within hundreds of proteins [18]. The readers facilitate signal propagation and formation of protein interaction networks. The ubiquitin signals can be edited or erased by deubiquitylating enzymes (DUBs) thus regulating the nature and duration of the signal [18]. These enzymes cleave the isopeptide bond at the end of a chain (exopeptidase activity) or within the polymer (endopeptidase activity). DUB activity screens have recently emerged as useful tools to examine native ubiquitin linkages associated with proteins [19,20]. There is a large field of study exploring how ubiquitin signals are read [21] and edited [22]. This review will instead focus on mechanisms underlying the assembly of ubiquitin signals.

**Figure 1:**
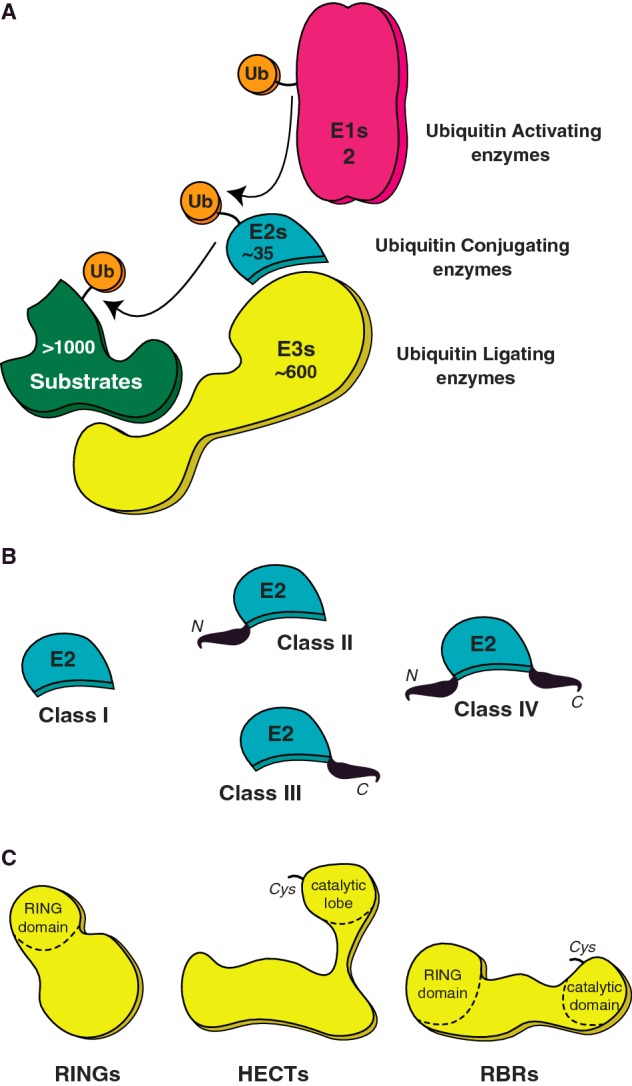
UBC pathway (**A**) A schematic of the ubiquitin (Ub) pathway and the members involved at each step; activation (E1), conjugation (E2) and ligation (E3). Ubiquitin activation is catalysed by the E1 in an energy-consuming step. The ubiquitin thioester conjugate is then passed onto the catalytic cysteine site on the E2 and finally ligated on to a target lysine of a substrate, an event mediated by E3 ligases. Also indicated is the numerical hierarchy of the ubiquitin pathway in humans. (**B**) Ubiquitin E2s are classified into four classes based on the N- or C- terminal extensions of the core UBC domain. (**C**) Three classes of E3 ubiquitin ligases; RINGs, HECTs and RBRs are classified based on their enzymatic mechanisms, with HECT and RBR ligase possessing a catalytic cysteine for Ub–E3 intermediate formation.

In humans, there are two E1s, 35 E2s and hundreds of E3s responsible for ubiquitination (Figure 1A). The E1s activate the C-terminus of ubiquitin and form an E1–ubiquitin thioester intermediate. The E2s collect the activated ubiquitin via a transthiolation reaction with the E1–ubiquitin thioester resulting in an E2–ubiquitin thioester intermediate. E3s either scaffold both the E2–ubiquitin thioester and the substrate to affect ubiquitination or form an E3–ubiquitin thioester intermediate prior to substrate conjugation. Recent advances in proteomics have generated an *in vivo* inventory of the ubiquitin system, thus providing a systems-level understanding of the pathway [23]. Similar PTMs can also occur through eight distinct ubiquitin-like proteins (Ubls), including the small ubiquitin-like modifier (SUMO) and the neural precursor cell expressed developmentally down-regulated protein 8 (NEDD8). Each of the Ubls has a similar, but smaller, cohort of proteins that facilitate the modification. The final steps of the cascade, i.e. cross-talk between E2 and E3 enzymes, decide the nature and target of the modification. The substantial number of possible combinations that can occur between ubiquitin E2 and E3 ligases supports a wide repertoire of ubiquitin signals across numerous substrates.

The activation of the C-terminus of ubiquitin by the E1 is the only energy-consuming step of the pathway. Structure-function studies on ubiquitin/Ubl E1s reveal multiple mechanistic details of the activation process. Briefly, the E1s have a multidomain architecture; the adenylation domain (binds Mg^2+^/ATP and the ubiquitin/Ubl), catalytic cysteine domain and the ubiquitin-fold domain (UFD, E2 selection) [24–27]. A long flexible linker (‘cross-over loop’) that connects the active adenylation domain with the catalytic cysteine domain bears crucial residues involved in ubiquitin/Ubl discrimination by the E1s [28]. Furthermore, a β-sheet on the UFD supports interactions with the E2 [25,29]. However, this surface is blocked until the formation of a ternary E1 complex (loading of both adenylate and thioester intermediates of ubiquitin/Ubl) that is ready to offload the ubiquitin/Ubl [30,31]. Conformational rearrangement in the E1 then facilitates the E2 catalytic cysteine to attack the E1 ubiquitin/Ubl thioester resulting in E2 loading (E2–ubiquitin/Ubl) [32,33]. Ubiquitin can be activated by two distinct E1s, ubiquitin activating enzymes (UBA) 1 and 6. Whereas UBA1 can load majority of the ubiquitin E2s, UBA6 functions with smaller set and is the sole E1 for the E2 enzyme Ube2Z [34–36].

The E2 conjugating enzymes share a core ubiquitin conjugation (UBC) fold, along with possible N-terminal and C-terminal extensions (Figure 1B) [37,38]. The UBC domain comprises an N-terminal helix, followed by a four-stranded meander that is surrounded by three α-helices. Tethered to the β-meander is a ‘flap-like’ β-structure that bears the catalytic cysteine, supported by a conserved HPN motif (histidine, proline, asparagine). In the ubiquitin/Ubl loaded E2 intermediate the ubiquitin/Ubl tail packs into the β-flap and the HPN motif asparagine supports ubiquitin conjugation of the target lysine [39–42]. In addition, residues surrounding the catalytic cysteine influence reactivity of ubiquitin loaded E2 with thiol or amine acceptors [43]. Beyond the active site, the N-terminal surface on the UBC fold (helix1, Loops 1 and 2) scaffolds both E1 and E3 interactions. This ensures E1–E2 and E2–E3 binding events are mutually exclusive and regulate the flow of the pathway [29,44–46]. The interactions between E2 and E3 are usually weak and transient in nature. Structures of E2–E3 complexes reveal how this E2 surface affords plasticity [47–49] as well as specificity [50] in its E3 interactions. A sub-set of E2s have intrinsic mechanisms for building linkage specific polyubiquitin chains (Lys^11^, Lys^48^ and Lys^63^ linked) using distinct non-covalent interactions with ubiquitin [51–54]. A further E2 surface is the ‘backside’ on the β-meander opposite the active site. On certain E2s, this surface supports non-covalent interactions with ubiquitin/Ubls thereby enhancing chain formation via E3-independent [55,56] and E3-dependent mechanisms [57].

Finally, there are three classes of E3 ubiquitin ligases; homologous to the E6–AP C-terminus (HECTs) [58], really interesting new genes (RINGs) [59] and RBRs [60], classified based on their enzymatic mechanisms. The RING domain ligases, the largest of E3 ligase family (∼600 members), facilitate direct transfer of ubiquitin from the E2 to the substrate. They contain a RING domain (Figure 1C, left) that co-ordinates two zinc ions in a cross-brace topology [61–63]. The U-box domain, a RING variant, also adopts a cross-brace topology stabilized instead by a hydrogen-bonding network [64,65]. In RING domains, the loops bearing the first and last pair of zinc-binding sites together with a central helix create an E2 binding cleft on the RING surface [45]. Structures of E2–E3 complexes reveal conserved hydrophobic residues on both the RING and E2 surface participate at the interface [66]. Furthermore, RING-type E3s rely on the associated E2 to generate different ubiquitin signals. The transient nature of E3–E2 interactions allow for E2 switching between initial ubiquitin conjugation and subsequent chain elongation events [67–69]. In addition, RING domain E3s can function as monomers (e.g. FANCL [70], Cbls [71], RNF168 [72]), homodimers (e.g. RNF4 [73], cIAPs [74], CHIP [49]) or heterodimers (e.g. BRCA1/BARD1 [75], RING1b/Bmi1 [76], MDM2/MDMX [77]). Substrate recognition is varied among the E3s. They can occur directly through the RING and E2 domains as seen in the structure of the PCGF4/RING1B–UbcH5c complex bound to the nucleosome [78] or through flanking regions such as the N-terminal tyrosine kinase-binding domain of c-Cbl RING E3 ligases [71]. Recent structures of RING domain E3s in complex with ubiquitin loaded E2s (E2-Ub) [79–82] demonstrate how the E3 induces a ‘closed’ or active E2-Ub conformation, stabilized by additional E2–ubiquitin (Ile^44^ patch) and RING–ubiquitin (Ile^36^ path) contacts, thus priming the complex for ubiquitin conjugation. Additional non-RING based E2 interaction elements are found in some RING ligases. The RING E3s gp78 and Cue1p both contain distal helical domains, Ube2g2-binding region (G2BR) and Ubc7p-binding region (UB7R) respectively, which bind the E2s ‘backside’ surface. The backside binding of the helical domains have different allosteric effects at their respective E2 active sites [83,84] nevertheless, both enhance RING affinity and facilitate processive ubiquitination events [84,85].

In addition, RING domains also appear in multi-subunit protein complexes such as the Cullin RING ligase (CRL) family. The CRLs are a modular complex comprising an elongated cullin scaffold protein (six types) interacting with monomeric RINGs (Rbx1/Roc1/Hrt1) at the C-terminus and a wide range of substrate recognition modules (cullin adaptor proteins and substrate receptors) at the N-terminus [86]. CRLs adapt numerous substrate recognition modules to recognize specific target proteins thus resulting in over 500 distinct E3 ligase complexes. As the substrate and E2-binding sites reside on opposite ends of a CRL complex [87], a site-specific neddylation event induces a conformational release of the RING domain from Cullin's C-terminal domain, thereby activating the E3 for substrate ubiquitination [88,89]. The assembly and regulation of CRLs are reviewed elsewhere [90]. The anaphase promoting complex or cyclosome (APC/C) is another large multi-subunit cullin-RING ligase that contains 13 core subunits including a cullin-like scaffold (Apc2), a small RING protein (Apc11) and two co-activator subunits, Cdc20 and Cdh1, which recognize distinct substrates [91,92]. The CRLs and APC/C are active during different phases of the cell cycle and regulate critical cellular events through degradative ubiquitination. These functions are discussed in greater detail in other reviews [92,93].

The HECT domain ligase family (28 members) have a C-terminal catalytic HECT domain that forms a catalytic intermediate with ubiquitin prior to substrate modification [58,94]. The N-terminal extensions of the HECT domain carry out substrate recognition functions. The catalytic HECT domain has two structural ‘lobes’, the E2 binding N-lobe and catalytic cysteine bearing C-lobe, tethered by a flexible linker (Figure 1C, middle) [44,58]. Structures of the NEDD4-family HECT domains reveal how dramatic conformational changes orient the C-lobe towards the E2 docking site during ubiquitin loading and subsequently rotate the C-lobe–ubiquitin intermediate towards the substrate for ubiquitin conjugation events [95–97]. Furthermore, most HECT E3s have an intrinsic capacity to build ubiquitin chains that is independent of the E2 pairing [98,99]. Auto-inhibition is a notable feature of HECT E3s, mediated via intramolecular interactions between domains/motifs located on the N-terminal extensions and the HECT domain [100–103].

Finally, the RBR family (13 members) features a RING domain and also bears a catalytic cysteine that forms a thioester–ubiquitin intermediate (Figure 1C, right) [43]. The RBRs function through a unique two-step RING–HECT hybrid mechanism whereby interaction between a RING domain and the ubiquitin loaded E2 facilitates the ubiquitin loading of a catalytic domain. Thus, a HECT-like ubiquitin–thioester intermediate occurs prior to the substrate conjugation event. Notably, the interaction between the RING domain and the E2–ubiquitin thioester does not directly support substrate ubiquitin conjugation [104,105]. An interesting example of RBRs is the linear ubiquitin chain assembly complex (LUBAC) consisting of the haem- oxidized IRP2 ubiquitin ligase-1 (HOIL-1) isoform HOIL-1L, HOIL-1L interacting protein (HOIP) and SHARPIN [106–109]. The LUBAC complex is only E3 ligase that can synthesize linear polyubiquitin chains with a range of E2s. Interestingly, the catalytic cysteine domain of HOIP has an additional zinc finger that cooperates with a linear ubiquitin chain-determining domain (LDD) to position the acceptor ubiquitin during linear ubiquitin chain formation [110]. Structural and enzymatic features of RBRs, in particular Parkin, will be discussed in more detail later in this review.

Hundreds of E3 ligases confer specificity of ubiquitination and play crucial roles in almost every cellular process. Unsurprisingly, deregulation of these enzymes is linked to several human diseases. Mutations in Mdm2 [111], Von Hippel–Lindau (VHL) [112], BRCA1 [113], TRIMs [114] and other E3s have been linked to multiple cancers. Further, deregulation of E3 ligases such as Parkin and E6–AP are linked to Parkinson's disease (PD) [115] and Angelman syndrome [116] respectively. Understanding the molecular mechanisms of ubiquitin signal assembly requires a biochemical and structural understanding of the event. We are focused on understanding specificity in the ubiquitin system, at every level. This includes specificity of the pathway components, specificity for substrates and target lysines and type of modification. To address these questions we use model systems that represent opposite ends of the spectrum of specificity. One system, the Fanconi Anaemia (FA) DNA repair pathway, has one modification, one target lysine, one E3–E2 pair. A second system has a broad spectrum of targets, modifications and components. Importantly, both systems have broad significance in fundamental biology and disease settings and the remainder of this review describes our contributions to answering these questions.

## FANCL: a selective and specific E3 ligase mutated in Fanconi anemia

The multi-step process of DNA damage repair relies on distinct ubiquitin signals to co-ordinate the damage response. Several RING E3 ligases play crucial roles in these pathways [117]. DNA inter-strand cross-link (ICL) repair is one such example where a specific monoubiquitin signal is required for the recruitment of repair factors [118]. ICLs are lethal lesions that block strand separation during DNA replication and transcription. The damage can be induced by various environmental or chemical mutagens and the toxicity of intercalating agents is widely exploited during chemotherapy [119]. The FA pathway is required for ICL repair [120]. Mutations in this pathway give rise to FA, a devastating childhood genome instability disorder, typified by bone marrow failure and a high predisposition to cancers [121,122]. FA patient cells are highly susceptible to ICL mutagens and display higher levels of chromosomal abnormalities [123].

**Figure 2:**
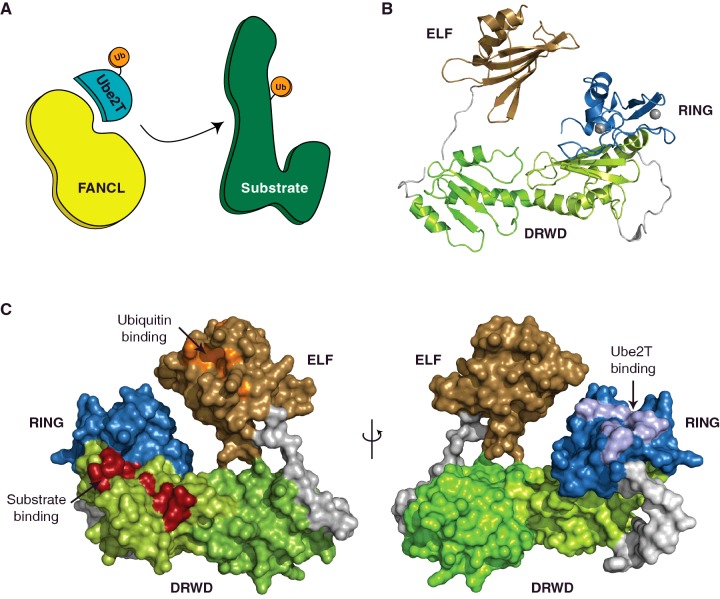
Specificity of the E3 ligase FANCL (**A**) Schematic of the FANCL and Ube2T-mediated specific substrate monoubiquitination. (**B**) Ribbon diagram depicting the FANCL structure (PDB 3K1L). The ELF, DRWD and RING domains are coloured light brown, green/lime and blue respectively. Zinc atoms are represented as grey spheres. (**C**) Surface representation of the protein interaction surfaces on FANCL. The binding patch for ubiquitin (orange) and substrate (red) reside on the ELF and DRWD domains respectively (left). The E2 binding surface (light blue) is on the RING domain (right).

Eighteen proteins (FANC-A–C, D1, D2, E to G, I, J, L to Q, S and T) along with several FA-associated proteins (FAAPs) participate in the FA pathway. Mutations in any of the FANC genes are linked to a failure in ICL repair [124–127]. A critical pathway signal is the site-specific monoubiquitination of FANCD2 and to a lesser extent a structurally homologous protein FANCI [128–131]. A large, nuclear, multi-protein core complex regulates the monoubiquitination event [132]. The core complex comprises eight FANC proteins (FANC-A, B, C, E, F, G, L and M) and five FAAPs (FAAPs 10, 16, 20, 24 and 100). FANCL has a RING domain and is the E3 ligase subunit of the FA core complex that functions with Ube2T, the E2 for the FA pathway [133,134]. Mutations in Ube2T have recently been linked to a FA phenotype, which is now denoted as FANCT [125–127]. The exquisite specificity of this ligase ensures the strict monoubiquitination of a single lysine on two related substrates thus making it an attractive model system to understand the underlying mechanisms of ubiquitination (Figure 2A).

FANCL was initially predicted to contain three WD (tryptophan-aspartate dipeptide) 40 repeats and a C-terminal plant homoeodomain (PHD) zinc-finger [135,136]. A FANCL fragment containing the PHD zinc-finger was capable of *in vitro* auto-ubiquitination. However, PHD zinc-fingers are not associated with ubiquitin E3 ligase activity [137] suggesting that FANCL may not harbour a PHD. Further, the loss of FANCD2 monoubiquitination in FANCL-null cells can be rescued by ectopic expression of wild-type FANCL, but not by zinc-finger mutants, confirming its E3 ligase activity [136]. Studies also put forth a role for FANCL WD40 repeats in mediating core complex interactions, whereas FANCE was linked to substrate recognition [138,139]. These observations together suggest a modular architecture for the FA core complex, similar to multi-subunit E3 ligase complexes [66], where substrate binding and ubiquitin ligase activity are undertaken by different subunits. However, invertebrates (fly, worms and slime moulds) have an apparently simpler system comprising FANCM, FANCL and FANCD2 and FANCI [140–143]. This suggests, at least in early evolution, FANCL is sufficient for both substrate recognition and the ubiquitination events. Furthermore, biochemical reconstitution of FANCD2 monoubiquitination using only recombinant chicken FANCL and Ube2T indicates that the rest of the core complex is not needed *in vitro* [144]. The same study also identified a RWD fold [145] in the predicted WD40 repeat region. This suggests FANCL has a different molecular architecture to that originally predicted.

In order to understand how FANCL functions we set out to structurally characterize the E3 ligase. Numerous attempts to express the full-length human FANCL using various solubility tags and expression systems yielded little success. Subsequently, we shifted our efforts to FANCL homologues from invertebrates that appear to have the minimal FA pathway components. We successfully expressed and purified *Drosophila melanogaster* (*Dm*) FANCL, which shares ∼20% sequence identities with human FANCL. This proved crucial to our success as we could purify *Dm*FANCL and obtain diffraction quality crystals. The resulting structure, refined to 3.2 Å (1 Å=0.1 nm; PDB 3K1L), revealed a remarkably different molecular architecture of FANCL to that predicted. FANCL comprises three domains (Figure 2B), an N-terminal E2-like fold (ELF), a novel double-RWD (DRWD) and a C-terminal RING domain [70]. Given the unexpected architecture, our first question was which domain, if any, supports substrate binding. *In vitro* pull-down analyses show that FANCL fragments bearing the DRWD–RING are necessary and sufficient to establish the binding of substrates FANCD2 and FANCI. The FANCL structure is extended, in particular, the ELF domain makes no contacts with the rest of the protein. The ELF domain bears the β-meander found in all E2 enzymes. However, instead of a catalytic cysteine β-flap, it has a fifth strand that packs against the meander [70]. Interestingly, we find an exposed surface of the ELF domain to interact non-covalently with the Ile^44^-hydrophobic patch of ubiquitin [146]. Several E2s also support non-covalent interactions with ubiquitin/Ubls [55,147,148]; however, the ELF surface involved (Figure 2C) is distinct from the ‘backside’ surface used by E2s. The ELF residues at this region share weak sequence homology across FANCL species, yet retain ubiquitin-binding, indicating a conserved functional role. In cells, mutation of this ubiquitin binding patch of FANCL impairs the monoubiquitination of both FANCD2 and FANCI suggesting another layer of regulation in the FA pathway [146].

The DRWD domain was a surprise on several levels. First, it is made up of RWD repeats (found in three major families: RING-containing proteins, WD-repeat-containing proteins and yeast DExD-like helicases [145]) and not of WD40 blades. Two RWD folds are linked via a long kinked helix to form the DRWD domain. This domain has a compact structure, with a continuous hydrophobic core and neither lobe could be expressed separately. Furthermore, a DALI search with the DRWD domain yielded no structural homologues suggesting a novel domain [70]. Second, the DRWD domain is required for substrate recognition. We also determined the structure of human DRWD, resolved to 2.0 Å (PDB 3ZQS) [149]. It shares the bilobal architecture as the *Dm*DRWD domain but with a β-element in the N-terminal lobe, helical in *Dm*DRWD, and hence more similar to a UBC fold. Analysis of solvent-exposed residues of human DWRD reveals several hydrophobic patches conserved between the human and fly proteins [132]. Mutation of these patches reveals that lobe2 is the substrate-binding domain [132] and induces substrate monoubiquitination [150] (Figure 2C). Notably, the isolated DRWD domain is sufficient to interact with substrates FANCD2 and FANCI. The fact that mutations of surface exposed residues affect FANCL interactions with both FANCD2 and FANCI suggests that substrate specificity is driven by FANCL [149].

Structural and biophysical studies of other protein complexes have revealed how different RWD domain arrangements (homo/hetero dimers or as tandem repeats) are integral building blocks of multi-protein assemblies. Subsequent to our discovery of the DRWD domain, the same double-RWD architecture has been observed in multi-subunit protein complexes at both the inner and the outer kinetochore [151–153]. Within the ubiquitin conjugation pathway, single RWD domains have been linked to enhancing E2 activity [154,155] and for positioning the E3 ligase Listerin at the 60S ribosome for degradative ubiquitination of stalled translation products [156]. Curiously, RWD domains are predicted in several E3 ligase proteins, however we currently have limited appreciation for their roles in regulating ubiquitination.

The inherent substrate specificity exhibited by FANCL helps explain how the E2–E3 pair of Ube2T–FANCL mediates FANCD2 monoubiquitination in absence of the FA core complex [144]. Interestingly, analytical size-exclusion chromatography reveals that Ube2T forms a stable complex with the isolated FANCL RING domain [149]. Similar experiments between the RING domain and several different E2s (Ube2-B, D3, H, K, L3, L6 and R1) show no complex formation [50]. Furthermore, the FANCL RING can selectively complex with Ube2T from a pool of E2s. Interactions between RINGs and E2s are generally of low affinity (high micromolar range) and typically involve conserved, hydrophobic side chains (for example, Ile^309^ and Trp^341^ on FANCL RING domain and Phe^63^ on Ube2T) [66]. Mutation of the conserved hydrophobic E2 residue (Phe^63^) results in a significant binding defect (>10 fold) in RING–Ube2T interaction [149]. Indeed, only a FANCL–Ube2t pair results in the site-specific monoubiquitination of FANCD2 and the pair form a tighter complex than other E3–E2 pairs [50].

In order to understand the molecular basis of the apparent E3–E2 specificity, we set out to structurally characterize the FANCL–Ube2T complex. Our initial attempts to crystallize the RING–Ube2T complex resulted in poorly diffracting (∼11 Å) crystals. Despite numerous efforts we could not improve the crystals. As an alternative strategy, we expressed and purified a RING–Ube2T fusion protein bearing a short and flexible linker sequence between the E3 and E2 domains. The E3–E2 chimera successfully crystallized and diffracted to 2.4 Å (PDB 4CCG) [50]. Incidentally, this fusion strategy has been subsequently employed in resolving structures of other RING–E2 complexes [78,157]. In our FANCL RING–Ube2T structure, individual RING and E2 domains adopt similar folds to those observed in the isolated *Dm*FANCL (PDB 3K1L) and Ube2T (PDB 1YH2) structures respectively. Thus, no major conformational changes occur during RING–E2 complex formation. The buried interface area in our structure (∼700 Å^2^) however, is markedly greater than other RING–E2 complexes (PDBs 2YHO, 3EB6, 3RPG, 4AUQ, 4ORH, 4V3K and 5AIE interface area range: 450–600 Å^2^). Accordingly, our structure reveals the RING–E2 interactions are extended beyond the generic E3–E2 interface to include an extended hydrophobic interface and an extensive network of polar and electrostatic contacts that stabilize the complex. In particular, Tyr^311^ of FANCL docks within an Ube2T pocket embraced by Arg^6^, Arg^9^ and Asn^103^ side chains. This residue is highly variable in other RINGs and is absent from the canonical RING–E2 interface. Furthermore, a critical basic residue on Ube2T (Arg^60^), predominantly acidic in other E2s, forms a salt bridge with Glu^340^ of FANCL serving as the positive selector for the FANCL RING–Ube2T pairing. Mutating residues at the canonical RING–E2 interface, as well as those unique to our structure (Figure 2C) disrupts the FANCL RING–Ube2T complex and results in loss of FANCD2 monoubiquitination.

Our FANCL–Ube2T structure also offers insights into the site-specific substrate monoubiquitination events. A shared E2 interaction surface is required for both ubiquitin loading via the E1 and offloading via the E3 [46]. Following monoubiquitination of FANCD2/I, the low Ube2T off-rate exhibited by FANCL can limit E2 dissociation and thus prevent ubiquitin reloading of the E2 and recurrent ubiquitination. In a related scenario, the E3–E2 complex can specify the FANCD2/I surface that exhibits the target lysine. Site-specific monoubiquitination can then occlude this surface from subsequent FANCL–Ube2T recognition. Our RING–Ube2T structure also reveals pockets outside of the generic E3–E2 interface for designing small molecules that could selectively interfere with the pair. The targeted disruption of the FANCL–Ube2T complex, when used in conjunction with chemotherapy, would inhibit FANCD2 and FANCI ubiquitination and hence the ICL repair pathway, thereby enhancing efficacy of chemotherapeutics.

E3 interactions with E2 govern the type of the ubiquitin signal that is conjugated on substrates and this is critical for the downstream outcomes of the signal. Our work on structure-function characterization of FANCL reveals how this E3 encodes inherent specificity for its substrates FANCD2 and FANCI. Further, we uncover the selective features on FANCL RING that control interactions with its physiological E2 partner Ube2T. This exquisite selectivity determines how the correct ubiquitin signal is generated for the ICL repair pathway to progress.

## Parkin: a broad-spectrum promiscuous E3 ligase mutated in Parkinson's disease

**Figure 3:**
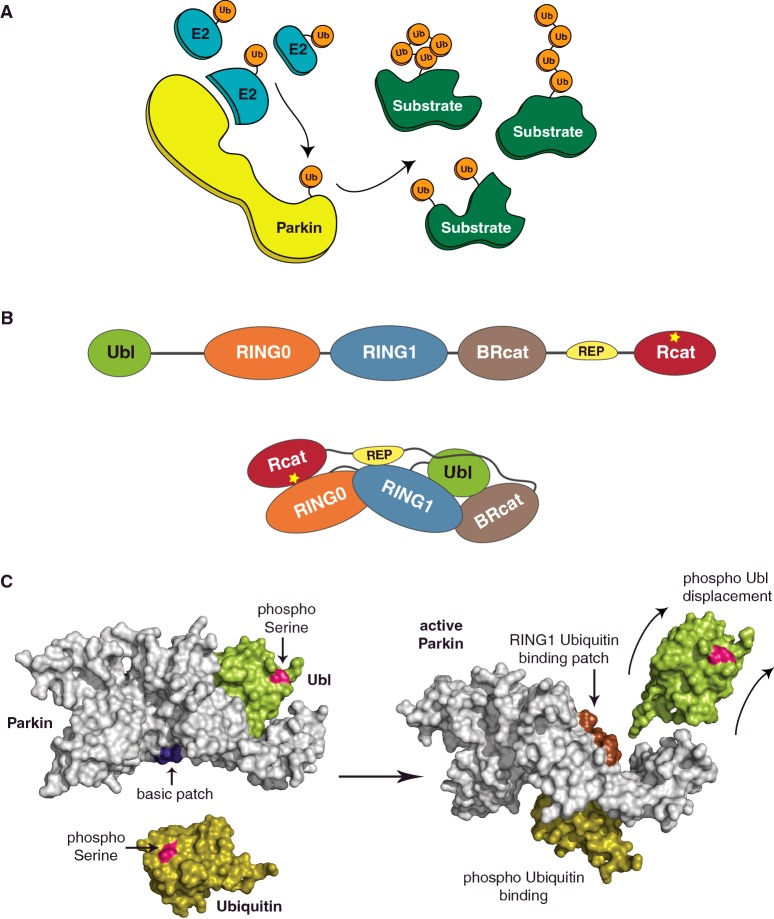
The broad-spectrum E3 ligase Parkin (A) Schematic of the variety of substrate ubiquitination events mediated by Parkin through several E2 enzymes. (**B**) A cartoon depicting the domain/motif arrangement of full-length Parkin (top) and the multiple inter-domain interactions that stabilize the tertiary structure (bottom). The RBR module comprises RING1, BRcat and Rcat domains with the catalytic cysteine (yellow star) present in the Rcat domain. Additional regulatory domains/motifs are the Ubl domain, a zinc-chelating RING0 domain and the small helical REP. (**C**) Surface representation of Parkin (grey, PDB 5C23) shows the distal location of phospho-serine (pink) on the Ubl (lime) domain and basic patch (dark blue) created on the surface of the RING0/RING1 interface (left). Binding of phospho-ubiquitin (orange, PDB 4WZP) to the basic patch on Parkin (right) leads to the complete displacement of the phospho-Ubl domain exposing the ubiquitin-binding patch on Parkin's RING1 domain (brown). This exposed patch on Parkin can support interactions with multiple E2–Ub intermediates and hence catalyse diverse ubiquitin signals.

A second model for understanding specificity in ubiquitination is Parkin, an E3 ligase mutated in heritable forms of PD. As an E3 ligase, Parkin is reported to have hundreds of putative targets including itself, can function with multiple E2 enzymes and is apparently capable of effecting multiple types of ubiquitin signals [158] (Figure 3A).

PD is the second most prevalent neurological disorder. PD affects approximately 1% of the population above the age of 50, with 5% of the cases being rare familial forms with an earlier onset (<45 years). Symptoms include bradykinesia, resting tremor and muscular rigidity, associated with the progressive loss of dopaminergic neurons in the substantia nigra. Research in the last two decades has uncovered multiple genetic causes underlying what was previously considered to be sporadic disease [159–161]. Linkage and genotype analyses of familial PD cases have identified a subset of genes, including PARK2 and PTEN induced putative kinase (PINK)1, associated with autosomal-recessive patterns of inheritance [162]. Mutations in PARK2, which encodes the RBR E3 ligase Parkin, are linked to nearly half of the recessive early-onset PD cases [163,164]. In addition mutations in PARK2 are also found in several cancer states [165,166]. Parkin belongs to the RBR family of E3 ligases, which were originally classified due to inclusion of two predicted RING domains (RING1 and RING2) separated by an in-between-RING domain, collectively termed the ‘RBR’ module [167]. However, a solution structure of the RING2 domain from HHARI suggested that RING2 did not adopt a canonical RING fold [168] and recent structures of RING2 domains from RBR proteins HHARI [104,169], Parkin [170–172] and HOIP [110] reveal that RING2 is not a RING domain at all. In fact, the RING2 domain is a linear zinc-chelating domain, which bears a cysteine required for catalysis [43] and adopts the same fold as the ‘in-between-RING’ domain [173]. The in-between-RING domain is neither between RINGs nor bears a catalytic residue; therefore, we refer to the domains as RING1, required-for-catalysis (Rcat) and ‘Benign’ Rcat (BRcat) to retain the RBR nomenclature [166]. There are 13 eukaryal RBR proteins and they all have varied domains outwith the common RBR module [167]. Parkin has an N-terminal Ubl domain and a zinc-chelating RING0 domain [174] additional to the RBR (Figure 3B). Importantly, at least 80 pathogenic amino acid substitutions that lead to autosomal recessive PD are found throughout the primary sequence of Parkin, clustering in domains, but also in the linkers between domains [175]. We set out to determine the structure of Parkin in order to understand its apparent promiscuity, mechanism of ligase activity and how disease mutations affected its function. As with any crystallographic project, our first challenge was to produce large quantities of stable and pure protein. To achieve solubility we fused the small ubiquitin-like modifier, Smt3 (suppressor of MIF2, SUMO in mammals) to the N-terminus of Parkin [176]. Removal of Smt3 is achieved via a SUMO-specific protease, ubiquitin-like-specific protease (Ulp)1 [177], which recognizes the tertiary fold of Smt3 and cleaves at the exact C-terminus, leaving no overhang or leader sequence. However, the purified recombinant protein lacked ubiquitination activity. Historically, auto-ubiquitination assays are used to assess the E3 ligase potential of a protein [1,178,179]. Parkin was reported to be a constitutively active E3 ligase [180,181] and the auto-ubiquitination readout was extensively used to characterize the effects of its pathogenic mutations in Parkin [182–186]. Despite extensive efforts to reproduce the assays reported by many others, our Parkin preparations were not active for auto-ubiquitination. Puzzled by the apparent lack of activity, we noticed that a common feature among all ‘active’ Parkin reports was the presence of epitope or solubility tags at the N-terminus of the protein. Thus, we assayed the fusion protein for activity and found to our surprise that it was competent for auto-ubiquitination activity. Indeed, we fused multiple tags to the N-terminus of Parkin and found that when tagged, Parkin was capable of auto-ubiquitination and removal of the tag rendered Parkin inactive [187,188]. These data suggested Parkin activity was linked to perturbations of its N-terminus. Interestingly, Parkin's Ubl domain bears several pathogenic mutations that influence its stability and is also involved in mediating putative substrate ubiquitination and proteasomal interactions [189–194]. Surprisingly, deletion of the Ubl domain species dramatically improves its auto-ubiquitination activity [187]. Furthermore, multiple pathogenic point mutants within the Ubl domain also trigger auto-ubiquitination both *in vitro* and *in vivo*. Thus, wild-type Parkin appears to be a dormant E3 ligase that is inhibited by a native Ubl domain. A series of experiments further uncovered an intramolecular interaction between an Ile^44^-centred surface on the Ubl domain and the rest of molecule. Structurally similar to ubiquitin, the Ubl domain also boasts an Ile^44^ hydrophobic surface. Interestingly, we find this ubiquitin surface to be required for auto-ubiquitination and Parkin variants with compromised intramolecular states enhance offloading of the E2–ubiquitin intermediate. The auto-regulation exerted by the Ubl domain thus involves the obscuring of catalytic interactions between Parkin and the ubiquitin loaded E2 [187].

Our finding that Parkin is an auto-inhibited protein and hence constitutively inactive was not initially met with universal enthusiasm, despite several cell-based observations that hinted at a ‘latency’ in Parkin that required activation [195–197]. A growing body of evidence had revealed roles for PINK1, a kinase also mutated in autosomal recessive PD, and Parkin in mitophagy, whereby damaged mitochondria are processed via ubiquitination and subsequent autophagic clearance [198,199]. Interestingly, the kinase activity of PINK1 is required for Parkin translocation to damaged mitochondria and was also suggested to activate its E3 ligase potential [195–197]. A direct functional link was uncovered when PINK1 was reported to phosphorylate Parkin at Ser^65^ in the Ubl domain, leading to Parkin activation [200,201]. In fact, activated Parkin triggers ubiquitination on dozens of different mitochondrial proteins with various polyubiquitin signals (Lys^27^, Lys^48^ and Lys^63^-linked chains) [202–205]. An earlier breakthrough study describing the RING–HECT hybrid mechanism for all RBR ligases could not capture the Parkin Cys^431^–ubiquitin thioester intermediate [43] and a number of studies subsequently showed that both mitochondrial damage and the kinase activity of PINK1 is required to induce the Parkin–ubiquitin intermediate. In addition, multiple E2s can trigger the ubiquitin charging and consequent ubiquitination of mitochondrial proteins by Parkin [206–208]. Thus a consensus emerged of PINK1–Parkin cross-talk as a pre-requisite for mitochondrial homoeostasis.

At this point, we understood that Parkin was auto-inhibited and could be activated by phosphorylation of the Ubl domain by PINK1. We also knew that pathogenic mutations in the Ubl domain lead to constitutively active Parkin and that the number of putative Parkin substrates was increasing rapidly. The inheritance of Parkin-related Parkinsonism is currently accepted to be autosomal recessive [164]. There are PD cases where the patient is a compound heterozygote and one question our observations immediately provoked is how activating mutations lead to a recessive inheritance when ‘gain-of-function’ mutations might be expected to be dominant? We hypothesized that activating Parkin mutations lead to self-ubiquitination and subsequent degradation, thus resulting ultimately in loss of Parkin. We tested our theory through a series of *in vitro* and cell-based experiments. What we found is that not only are Parkin mutants rapidly degraded in cells, but that Parkin can only ubiquitinate Parkin in *cis*, not in *trans*. In other words, an active pathogenic mutant of Parkin only ubiquitinates itself, but does not modify another molecule of Parkin. In the heterozygous context, the mutant Parkin (unstable, active or inactive) would regulate its own status without influencing another copy, thus clarifying the recessive phenotype linked with the majority of pathogenic Parkin mutants [169].

In 2013, several groups reported the structure of the RING0–RING1–BRcat–Rcat (R0RBR) domains of Parkin (residues 141–465) [170–172]. These structures reveal a complex arrangement of the four domains (Figure 3B), with the interface between the R0–Rcat domains reportedly occluding the catalytic cysteine (Cys^431^) and a small helical element, termed repressor element of Parkin (REP) that packs against the predicted E2-binding site. Thus, even in the absence of the first 140 amino acids, including the Ubl domain, Parkin adopts an apparently auto-inhibited conformation. Biochemical and structural studies of several multi-domain RBRs reveal auto-inhibition to be a characteristic feature of this enzyme family [104,105,170–172,209,210]. Although these structures revealed the compact and interdependent nature of Parkin domains and explained the molecular basis of many pathogenic mutations, it was still not clear how the Ubl domain inhibits Parkin or how activation could be achieved. In 2014, an exciting new regulatory layer within the PINK1–Parkin and mitophagy pathway was revealed. In addition to Parkin Ser^65^ phosphorylation, PINK1 directly phosphorylates ubiquitin at an equivalent Ser^65^ residue, thus uncovering an unprecedented functional link between phosphorylation and ubiquitination pathways [211–214]. The dual Parkin–Ubiquitin phosphorylation events are both required for optimal Parkin activation and for amplifying ubiquitin signals on damaged mitochondria. Importantly, phospho-ubiquitin activates Parkin E3 ligase activity. PINK1 phosphorylation of pre-existing ubiquitin on the mitochondria also triggers the mitochondrial translocation and activation of Parkin. Recurrent PINK1 phosphorylation of ubiquitin signals mediated by active Parkin augments the entire cycle, leading to suggestions of a feed-forward mechanism [211,214]. Quantitative ubiquitin proteomics further reveal that the dual Parkin–Ubiquitin phosphorylation events generate diverse polyubiquitin signals (Lys^6^, Lys^11^, Lys^48^ and Lys^63^-linked chains) on damaged mitochondria. In fact, activated Parkin is catalytically productive with nearly two-dozen E2s *in vitro* and ubiquitinates numerous outer mitochondrial membrane proteins in cells (>30 high confidence targets) [205,214,215].

In order to understand the mechanisms of Parkin inhibition and activation, we needed to understand the molecular details of how the Ubl domain maintains the auto-regulated conformation and how the dual phosphorylation events offset this confirmation. Numerous crystallization trials with human full-length Parkin failed, however both the Ubl domain and the R0RBR region can be independently crystallized [170–172,216]. The Ubl–RING0 linker is poorly conserved across species, highly susceptible to proteolysis and not visible in a low-resolution structure of full-length rat Parkin [171,174]. Thus in order to crystallize all five domains, we removed this linker. UblR0RBR (Ubl residues 1–83 linked to R0RBR residues 144–465) displays an E3 ligase activity profile similar to full-length Parkin, but in contrast yields high-quality crystals that diffract to 1.8 Å (PDB 5C1Z) [217]. The refined UblR0RBR structure reveals a similar global structure to the R0RBR structures and in addition, the Ubl domain that packs tightly against the RING1 domain (Figure 3B). An extensive interface is formed, the largest domain/domain interface in Parkin. Notably the Ubl Ile^44^-centred surface is central to the interface, including many of the residues we previously observed to be activating when mutated [187]. However, we also observe a previously overlooked interface, burying 730 Å^2^ surface area, between the RING0–RING1 domains. In the structures of Parkin lacking the Ubl domain, this distant site is remodelled when compared with intact Parkin. In particular, a trio of residues His227-glu300-His302, have side chains pointing north towards the interior of the protein when the Ubl is present, which diametrically flip to point south towards the surface of Parkin when the Ubl is absent. Using isothermal titration calorimetry, we had previously observed stable interactions in *trans* between the Ubl domain and the rest of Parkin (residues 77–465) whereas activating Ubl mutants weaken this connection [187]. Using a similar setup we examined how the Parkin R0RBR interacts with different Ser^65^ variants of the Ubl domain and ubiquitin. The binding of Ubl to R0RBR, regardless of Ser^65^ status, is an exothermic process driven by negative enthalpic and small entropic changes. However, altering the Ubl Ser^65^ side chain diminishes the R0RBR binding event (∼2-fold with Ser65Asp/Glu and ∼10-fold with phospho-Ser^65^). Interestingly, interactions between ubiquitin (wild-type and Ser^65^ variants) and R0RBR have endothermic signatures. In particular, phospho-ubiquitin binding with R0RBR binding is driven by large positive changes in enthalpy (+32 kJ/mol) and entropy (+261 J/mol°K), suggesting an increase in disorder of the system. Changes in ubiquitin Ser^65^ also dramatically improve its affinity with R0RBR (∼10 fold with Ser65Asp/Glu and ∼4000-fold with phospho-Ser^65^). Taken together, the affinity profiles suggest that although wild-type Ubl stabilises the tertiary conformation, Ser^65^ variants counter this effect. Furthermore, contrasting thermodynamic profiles of phospho-ubiquitin binding suggest it interacts with a distinct Parkin surface and alters its structural integrity [217].

In order to understand how changes at Ubl Ser^65^ lead to Parkin activation, we attempted to crystallize Ser^65^ variants of UblR0RBR (Ser65Asp/Glu or phospho-Ser^65^). Whereas the phospho-Ser^65^ UblR0RBR crystallization proved fruitless, we successfully obtained Ser65Asp UblR0RBR crystals and refined the structure to 2.4 Å (PDB 5C23). Superposition of Ser65Asp UblR0RBR and UblR0RBR (RMSD 0.58 Å) reveals no global conformational changes. Remarkably, however, inclusion of a negative charge at position 65 of the Ubl domain causes the His227-Glu300-His302 side chains in the RING0/RING1 interface to adopt the south facing orientation observed in the absence of the entire Ubl domain. This subtle remodelling serves to create a continuous basic patch at the RING0/RING1 interface (residues His^302^, Arg^305^ and Lys^151^), which is presented on the surface of Parkin. Our study, as well other independent studies, shows the phospho-ubiquitin binding patch to be at the RING0/RING1 interface of Parkin [217–220]. A recent study shows that binding of phospho-ubiquitin to the R0RBR fragment of insect Parkin (body louse) leads to destabilization of a kinked RING1 helix [220]. The destabilizing of the RING1 helix confirms the thermodynamic profile we observe during phospho–ubiquitin interactions with the human R0RBR [217]. These observations reveal a model for Parkin activation where Ser^65^ phosphorylation of the Ubl domain weakens its packing with RING1 domains thus optimizing the RING0/RING1 interface for phospho–ubiquitin interactions.

In an elegant series of competitive binding spectroscopy experiments carried out by Gary Shaw's laboratory, the consequence of phospho-ubiquitin binding was shown to be the displacement of the Ubl domain. First, the titration of R0RBR Parkin triggers a spectral peak transition of a labelled Ser65Glu Ubl species from an unbound to a bound state. Remarkably, these signals revert back to the unbound state when Ser65Glu ubiquitin is subsequently added into the system. In a reverse setup, titration of R0RBR Parkin reveals the unbound to bound transition of the labelled Ser65Glu ubiquitin species. However these signals are unaffected upon addition of Ser65Glu Ubl domain. These data show that R0RBR Parkin is unable to simultaneously bind both phospho-ubiquitin and the Ubl domain. Whereas phosphorylation of the Ubl domain optimizes the binding of phospho-ubiquitin, interaction with phospho-ubiquitin induces the complete displacement of the weakened phospho/Ubl interface from the RING1 surface (Figure 3C). Furthermore, phospho-Ubl cannot rebind Parkin until the phospho-ubiquitin is released, thus sustaining an activated E3 conformation.

In our UblR0RBR structure, the largest buried interface is formed between the Ubl domain and the rest of Parkin (∼2150 Å^2^). The complete release of phospho-Ubl is predicted to expose a large interaction area including a RING1 surface that was once bound to the Ile^44^-hydrophobic patch on the Ubl domain. We had previously shown that a similar Ile^44^-hydrophobic patch on ubiquitin was required for Parkin auto-ubiquitination [187]. Thus, the displaced phospho-Ubl could expose a ubiquitin-binding surface on Parkin that is required for productive interactions with the E2–ubiquitin intermediate (Figure 3C). Consistent with this, a phospho-Parkin–phospho-ubiquitin complex displays a ∼20-fold increase in affinity with the ubiquitin loaded E2 compared with the isolated E2. Further, this increase in affinity requires an intact RING1 helix1 surface [217]. These insights suggest a model for Parkin activation whereby the Ubl domain maintains Parkin in an auto-inhibited state in the absence of phospho-ubiquitin signals. Upon activation of PINK1, both Parkin and ubiquitin are phosphorylated at Ser^65^, giving rise to the allosteric displacement of the Ubl domain. This displacement, along with the presumed displacement of the REP creates the E2-binding surface, presents a ubiquitin-binding site that simultaneously recruits the E2–Ub intermediate. This model provides a molecular explanation for the apparent lack of E2 specificity displayed by Parkin, since association with the common denominator, ubiquitin, contributes to most of the binding energy. The ability to scaffold different E2s also explains how Parkin can support catalysis of diverse ubiquitin signals.

## Concluding remarks

Our studies show that achieving specificity within a given pathway can be established by specific interactions between the enzymatic components of the conjugation machinery, as seen in the exclusive FANCL–Ube2T interaction. By contrast, where a broad spectrum of modifications is required, this can be achieved through association of the conjugation machinery with the common denominator, ubiquitin, as seen in the case of Parkin. There are many outstanding questions to understanding the mechanisms governing substrate selection and lysine targeting. Importantly, we do not yet understand what makes a particular lysine and/or a particular substrate a good target for ubiquitination. Subunits and co-activators of the APC/C multi-subunit E3 ligase complex recognize short, conserved motifs (D [221] and KEN [222] boxes) on substrates leading to their ubiquitination [223–225]. Interactions between the RING and E2 subunits reduce the available radius for substrate lysines in the case of a disordered substrate [226]. Rbx1, a RING protein integral to cullin-RING ligases, supports neddylation of Cullin-1 via a substrate-driven optimization of the catalytic machinery [227], whereas in the case of HECT E3 ligases, conformational changes within the E3 itself determine lysine selection [97]. However, when it comes to specific targets such as FANCI and FANCD2, how the essential lysine is targeted is unclear. Does this specificity rely on interactions between FA proteins? Are there inhibitory interactions that prevent modification of nearby lysines? One notable absence in our understanding of ubiquitin signalling is a ‘consensus’ ubiquitination motif. Large-scale proteomic analyses of ubiquitination sites have revealed the extent of this challenge, with seemingly no lysine discrimination at the primary sequence level in the case of the CRLs [228]. Furthermore, the apparent promiscuity of Parkin suggests the possibility that ubiquitinated proteins are the primary target of Parkin activity. It is likely that multiple structures of specific and promiscuous ligases in action will be required to understand substrate specificity in full.

## Abbreviations

APC/C: anaphase promoting complex or cyclosome
BARD1: BRCA1 associated RING domain protein 1
Bmi1: B cell-specific Moloney murine leukaemia virus integration site 1
BRCA1: breast cancer gene 1
BRcat: benign required-for-catalysis
Cbl: casitas B-lineage lymphoma proto-oncogene
Cdc: cell-division cycle protein
Cdh: CDC20 homolog1
CHIP: C terminus of HSC70 interacting protein
cIAP: inhibitor of apoptosis
Cue1p: coupling of ubiquitin conjugation to endoplasmic reticulum degradation protein 1
CRL: Cullin RING ligase
DRWD: double-RWD
DUB: deubiquitylating enzyme
ELF: E2-like fold
E6-AP: human papillomavirus E6-associated protein
FA: Fanconi Anaemia
FAAP: FA-associated protein
gp78: glycoprotein 78
HECT: homologous to the E6–AP C-terminus
HOIL-1: haem- oxidized IRP2 ubiquitin ligase-1
HOIP: HOIL-1L interacting protein
HHARI: human homolog of Ariadne
Hrt1: high level expression reduces Ty3 transposition protein 1
ICL: inter-strand cross-link
LUBAC: linear ubiquitin chain assembly complex
MDM: mouse double minute 2 homolog
NEDD8: neural precursor cell expressed developmentally down-regulated protein 8
PCGF: polycomb group RING finger
PCNA: proliferating cell nuclear antigen
PD: Parkinson's disease
PHD: plant homoeodomain
PTM: post-translational modification
PINK1: PTEN induced putative kinase-1
Rbx1: RING-box protein 1
Roc1: regulator of cullins 1
R0RBR: RING0–RING1–BRcat—Rcat
Rcat: required-for-catalysis
REP: repressor element of Parkin
RNF: RING finger
RWD: domain in RING finger and WD repeat containing proteins and DEXD-like helicases
SHARPIN: shank-associated RH domain-interacting protein
Smt3: suppressor of MIF2 protein
SUMO: small ubiquitin-like modifier
TRIM: tripartite motif containing protein
Ube2: ubiquitin E2
UBC: ubiquitin conjugation
UBD: ubiquitin-binding domain
Ubl: ubiquitin-like protein
UFD: ubiquitin-fold domain
Ulp1: ubiquitin-like-specific protease-1
WD40: tryptophan-aspartate dipeptide repeat
